# Does the association of therapeutic exercise and supplementation with sucrosomial magnesium improve posture and balance and prevent the risk of new falls?

**DOI:** 10.1007/s40520-021-01977-x

**Published:** 2021-09-12

**Authors:** Dalila Scaturro, Fabio Vitagliani, Pietro Terrana, Sofia Tomasello, Lawrence Camarda, Giulia Letizia Mauro

**Affiliations:** 1 Department of Surgical, Oncological and Stomatological Disciplines, University of Palermo, Via del Vespro, 129, 90127 Palermo, Italy; 2 University of Verona, Verona, Italy

**Keywords:** Rehabilitation, Magnesium deficiency, Senile osteoporosis, Balance, Posture

## Abstract

**Background:**

Fracture of the proximal femur is the most feared complication of osteoporosis. Given the numerous physiological functions that magnesium performs in our body, in the literature there is a correlation between osteoporosis and low serum levels of magnesium.

**Aim:**

Evaluate the incidence of hypomagnesemia in patients with lateral fragility fracture of the proximal femur, the possible correlation between serum magnesium levels and fractures, and the effectiveness of supplementing Sucrosomial^®^ magnesium associated with therapeutic exercise on the outcome of these patients.

**Methods:**

We divided the study into two parts. In the first part, we assessed the preoperative incidence of hypomagnesemia in patients using a blood test. In the second part, patients with hypomagnesemia were divided, in the post-operative period, into two groups, who received, respectively, only therapeutic exercise or oral supplementation with sucrosomial magnesium associated with therapeutic exercise.

**Results:**

Half of the patients with fragility femoral fracture had hypomagnesemia, with a higher incidence of the subclinical form. From the comparison between the two groups, the T1 treatment group showed a significant improvement in blood levels of magnesium (2.11 ± 0.15 vs. 1.94 ± 0.11; *p *< 0.05), on the NRS scale (5.7 ± 0.81 vs. 6.6 ± 1.18; *p* < 0.05), the Tinetti scale (17.3 ± 1.15 vs. 15.2 ± 2.98; *p* < 0.05) and the SarQoL questionnaire (47.3 ± 5.21 vs. 44.9 ± 5.54; *p* < 0.05).

**Conclusions:**

More attention would be needed in the diagnosis and correction of subclinical hypomagnesemia and not just the simple and clinically evident one, including hypomagnesemia among the modifiable risk factors for osteoporosis.

## Introduction

The fracture of the proximal portion of the femur is the most feared complication of osteoporosis.

Along with vertebral fractures, they are defined as brittle fractures, as they can occur as a result of low-energy trauma as a result of reduced bone mass and changes in bone structure [1].

This type of fracture accounts for 83% of hospitalization causes in osteoporotic patients. They are associated with high morbidity and mortality, in fact their mortality at 1 year is about 30% and at 5 years about 50% [2–4].

Given their high incidence, special attention should be paid to modifiable risk factors (cigarette smoking, alcohol, reduced body mass index (BMI), physical inactivity, and nutritional deficiencies).

It has been observed that nutritional deficiencies play an important role in the pathogenesis of osteoporosis [5, 6]. Bone metabolism is closely related to physical exercise and blood levels of calcium and vitamin D. An important role in bone metabolism is also played by some electrolytes, such as magnesium, and vitamins, like vitamin K. Their deficiency can have negative effects on the health of the bone, in fact now in the literature a correlation is known between osteoporosis and low serum levels of magnesium and/ or vitamin K. Their integration seems to have a promising role in preserving bone health [7, 8].

Magnesium plays an essential role in the physiological functions of our organism (heart, brain, muscle, and skeleton) [9–11]. Its physiological range in a healthy adult is 20–28 g with a plasma concentration between 1.7 and 2.6 mg/dl. Within the organism, 99% is present at the intracellular level (60% in bones and 39% in muscles and soft tissues) and the remaining 1% in the extracellular environment [12]. The daily dietary intake of magnesium should reach 420 mg in men and 320 mg in women [9]. Magnesium homeostasis is closely related to the interaction between the intestine (responsible for the absorption of dietary magnesium), bone deposit, and urinary excretion [12]. Foods rich in magnesium include: nuts, seeds, fruit, vegetables, and whole grains [13].

Risk factors related to the onset of hypomagnesemia are: alcoholism, poorly controlled diabetes, malabsorption diseases (e.g., Crohn’s disease and coeliac disease), chronic renal insufficiency and drug use (e.g. antibiotic, chemotherapy, diuretics and proton pump inhibitors) [14].

Hypomagnesemia occurs mostly in the chronic subclinical form with blurred or absent symptoms and plasma magnesium concentrations in the standard [15]. Covers more than 70% of the population [16]. The subclinical form is defined as the presence of serum magnesium less than 2.0 mg/dl, associated with urinary excretion within 24 h of 40–80 mg/day. Free hypomagnesemia is defined by the presence of serum magnesium of less than 1.7 mg/dl [17].

In the case of hypomagnesemia, the ion is mobilized from the bone to restore its normal plasma concentration with reduced activity of osteoblasts and stimulation of osteoclastic activity by the release of pro-inflammatory cytokines (TNF-alpha, IL-1, and substance P) which promote bone resorption [18].

Magnesium also influences serum parathormone (PTH) levels. Low serum magnesium levels result in paradoxical inhibition of PTH synthesis and secretion and resistance of peripheral tissues to the action of PTH since intracellular magnesium is a cofactor of adenylate-cyclase. This results in hypocalcemia which is not very responsive to treatment with calcium and vitamin D but is susceptible to correction with magnesium supplementation [7].

Also, magnesium is involved in maintaining the integrity of the endothelial wall, a fundamental component for bone nutrition [7].

Magnesium also acts at the muscle level serum magnesium levels are significantly associated with the risk of sarcopenia in the elderly. It also intervenes in transmembrane transport, muscle contraction, and relaxation affecting muscle performance. It is also involved in energy metabolism, affecting basic mitochondrial functions, including the synthesis of ATP and the removal of reactive oxygen species. Poor availability of magnesium can lead to reduced mitochondrial efficiency and increased production of reactive oxygen species resulting in structural and functional deterioration of proteins, DNA, and other essential molecules, in addition to a pro-inflammatory state [19].

In this study, we evaluated the incidence of hypomagnesia in patients with lateral fragility fracture of the proximal portion of the femoral, the possible correlation between serum magnesium levels and fractures, and the effectiveness of Sucrosomial^®^ Magnesium supplementation associated with therapeutic exercise inpatients outcome.

Sucrosomial^®^ Magnesium is an innovative oral preparation of magnesium oxide, covered by phospholipids plus sucrester matrix and can be used as an alternative to common magnesium salts to improve magnesium supplementation effectiveness. Through the encapsulation of magnesium ions within a sucrosomal membrane, they can cross the gastric and intestinal environment and reach the bloodstream without interacting with the intestinal mucosa. All this allows increasing intestinal bioavailability and the bioavailability of magnesium [20].

## Materials and methods

An observational prospective study was conducted between April 2020 to February 2021 at the UOC of Rehabilitation of the Paolo Giaccone University Polyclinic in Palermo, in collaboration with the UOC of Orthopedics and Traumatology of the same hospital.

The study was approved by the Medical Ethical Committee of the University Hospital of Palermo, Italy (n° 4/2020); written informed consent was obtained from each patient in conformity with Helsinki Declaration.

The Inclusion Criteria were: (1) age over 70; (2) diagnosis of lateral fracture by the fragility of the proximal portion of the femur.

Patients with altered states of consciousness, politraumatized, and subjects with medial fractures due to fragility of the proximal portion of the femur were excluded. Medial fractures have been excluded from the study as they can disrupt retinacolary vessels originating from the medial and lateral circumflex artery. The interruption of these vessels would involve the risk of avascular necrosis and pseudoarthrosis that would require an osteosynthesis with prosthetic replacement of the head of the femur responsible in our opinion to invalidate the rehabilitation protocol, as well as that in turn, would need of longer patient recovery times.

The aim of the first part of the study was the evaluation of total magnesemia in these patients. For the determination of magnesemia, the cut-offs used were [17]: values below 1.7 mg/dl for free hypomagnesemia; values below 2 mg/dl for subclinical hypomagnesemia; values above 2 mg/dl for normal magnesemia. Of 168 patients admitted for lateral fracture from fragility, 75 patients were recruited that met all the criteria of inclusion. Of these only 36 patients had subclinical hypomagnesemia (*n* = 32) or frank hypomagnesemia (*n* = 4). Following a randomization process to ensure equal distribution between the two groups (except for the 4 patients with free hypomagnesemia included in the treatment group), we chose to divide patients with hypomagnesemia (subclinical or frank) into a first group called “treatment group”, composed of 17 patients subjected to a rehabilitation protocol in association with a magnesium supplement; and a second group, called “control group” composed of 19 patients who have undergone rehabilitation protocol only.

The rehabilitation protocol provided to these patients consisted of two phases. A first phase, defined as maximum protection, lasting 3 days was carried out immediately after surgery during the stay in the department of Orthopedics and Traumatology. The objectives of this phase were: rapid verticalization and prevention of bedside complications. A second phase, defined as controlled movement and minimum protection, was carried out under the DH regime at the UOC Rehabilitation, with a duration of 18 days. The goal was the general recovery of the locomotor system, stimulating the reactions of balance and posture to prevent the risk of a new fall, and the improvement of daily life activities (ADL). In this phase passive and active mobilization techniques were used, muscle pumping exercises, muscle strengthening exercises, respiratory gymnastics, and exercises for the recovery of the correct pattern of pace and gait.

Patients in treatment group took a sachet of Sucrosomial^®^Magnesium (Ultramag^®^, Pharmanutra, Pisa, Italy) with a dosage of 375 mg of elemental magnesium, in oral suspension dissolved in a glasswater at room temperature, for 30 days, from the first day after surgery until the control visit, 1 month after the surgical intervention (T1).In addition, since all patients tested had a condition of insufficiency/deficiency of vitamin D [21] at the time of admission, they were also provided with an additional 25,000 UI cholecalciferol oral supplement once every 15 days from the first day after surgery to restore Vit D levels.

Patients were assessed at the basal, within 48 h of surgery (T0) and 1 month after the end of our rehabilitation protocol (T1) by: blood dosage of magnesium and vitamin Dand administration of assessment scales such as the NRS scale, the Tinetti test, and the Sarqol questionnaire.

The primary endpoint of the second part was to assess changes in serum levels of magnesemia in the two groups. The secondary endpoints were the evaluation oftolerability of Sucrosomial^®^ Magnesium, pain reduction, through the NRS scale, the reduction of the risk of new falls, through the Tinetti test, and the improvement of the quality of life, through the Sarqol questionnaire.

The Tinetti test, or Performance Oriented Mobility Assessment (POMA), is a clinical test commonly used to determine the equilibrium abilities of a subject, static and dynamic. It consists of two sections: the first examines the static balance in sitting and standing position, has 9 items, with a maximum score of 16 points; the second examines the gait, which has 7 items, with a maximum score of 12 points. The maximum total score that can be obtained is 28 points and based on the score obtained, three categories of patients can be distinguished: non-walker (0–1 score), risk of falls (2–20 score), and low risk of falls (score > 20) [22].

The Sarqolquestionnaire is a self-administrable psychometric tool produced to estimate the quality of life in subjects with sarcopenia and evaluate the changes in time in this population. It consists of 22 questions with 55 total items, specific for strength and muscle mass. The maximum score is 100 points, where the higher results reflect a better quality of life. Items are organized into seven domains: mental and physical health, locomotion, body composition, functionality, ADL, pleasure, and fear activities [23].

The NRS scale is a quantitative assessment scale by which patients are asked to assess their pain on a defined scale, from 0 to 10, best reflecting the intensity of pain at that specific time [24].

### Statistical analysis

Using the sample size estimation formula below,

$$n\, = \,{ }\frac{{p_{1} \left( {100 - p_{1} } \right)\, + \,{ }p_{0} \left( {100 - p_{0} } \right)}}{{\left( {p_{1} - p_{0} } \right)^{2} }} \cdot f\left( {\alpha ,\beta } \right)$$

Considering α = 0.05 and β = 0.10, we obtained a result of 39. Given the proximity between the theoretical sample size (equal to 39) and the actual sample size (equal to 36), we can conclude and assume that both the reliability values (1 − α = 0.95) and the power values (1 − β = 0.80) are respected. The data obtained were indexed on an Excel sheet and analyzed with the statistical software R. For the statistical modeling we used the classical linear regression model to evaluate the effect net of any confounding variables. *p *values < 0.05 were considered statistically significant. Pearson’s correlation index was then used to assess whether the variations were indeed proportional and how significant the association was.

## Results

The general characteristics of the patients included in the study are given in Table 1. After an evaluation of 168 patients admitted for lateral fracture from fragility, 75 patients (26 (34.6%) men and 49 (65.4%) women) met the inclusion criteria and were recruited. The average age was 75 ± 2.5 years. 54.6% of the fractures (*n* = 41) involved the right femur, while the remaining 45.4% (*n* = 34) involved the left femur. At the time of the initial clinical evaluation, patients reported an average NRS scale of 7.4–1.43. The most common anatomical site of the fracture, found in 49.3% of cases (*n* = 37) was the pertrocanteric region, and all received surgical treatment of osteosynthesis with endomedullary nail. More than half of the patients recruited (69.4%) had more than 3 comorbidities. At the time of admission, all the patients recruited showed a picture of vitamin D insufficiency/deficiency, with average values of 19.2 ± 7.43 ng/dl.

**Table 1:** General characteristicsat baseline of 75 patients

Characteristics	
Anthtopometric characteristics	
Age, mean ± SD	
	75 ± 2.5
Gender, n° (%)	
Male	26 (34.6)
Female	49 (65.4)
Weight, mean ± DS	74.3 ± 7.6
BMI, mean ± DS	24.6 ± 5.3
Clinical characteristics	
Laterality, n° (%)	
Right	41 (54.6)
Left	34 (45.4)
NRS, mean ± DS	7.4 ± 1.43
Anatomical site of the fracture n° (%)	
Pertrochanteric	37 (49.3)
Intertrochanteric	22 (29.3)
Subtrochanteric	16 (21.4)
Type of surgery, n° (%)	
Intramedullary nail	75 (100)
Comorbidity, n° (%)	
None	0 (0)
1–2	23 (30.6)
≥ 3	42 (69.4)
Vitamin D, mean ± DS	19.2 ± 7.43
Magnesemia levels, mean ± SD	1.92 ± 0.41

**Fig. 1:**
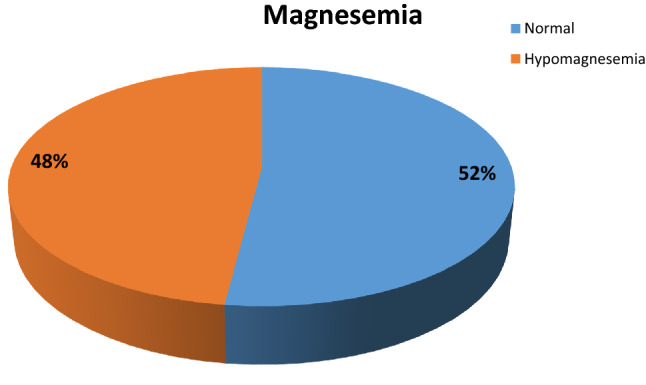
Magnesemia levels in patients at baseline

**Fig. 2:**
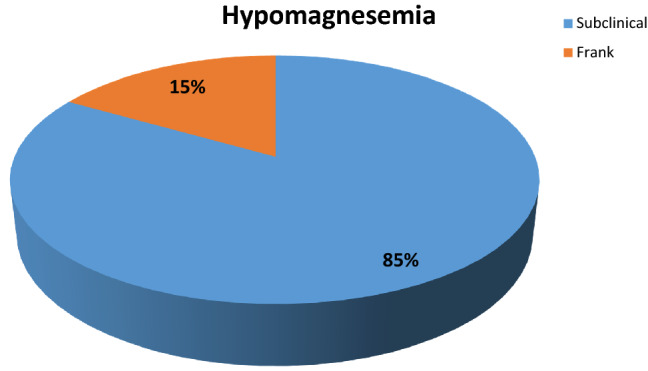
Prevalence of subclinical hypomagnesemia and frank hypomagnesemia in patients at baseline

Mean serum magnesium values before the surgery (T0) were 1.92 ± 0.41. 52% (*n* = 39) had normal magnesemia values, while the remaining 48% (*n* = 36) had preoperative hypomagnesemia (Fig. 1). Of these 85% (*n* = 30 patients) had subclinical hypomagnesemia, while 15% (*n* = 6) had frank hypomagnesemia (Fig. 2).

The comparison of the anthropometric and clinical characteristics at the base of the treatment and control groups is shown in detail in Table 2, where the homogeneity between the two groups is evident (*p* > 0.05).

**Table 2:** Characteristics at baseline of treatment group and control group

Characteristics	Treatment Group (*N* = 17)	Control Group (*N* = 19)	*p *value
Anthropometric characteristics			
Age, mean ± SD	71.2 ± 3.1	72.8 ± 2.7	0.16
Gender, n° (%)			
Male	6 (35.3)	5 (26.3)	0.43
Female	11 (64.7)	14 (73.7)	
Weight, mean ± DS	71.6 ± 4.6	74.2 ± 2.4	0.34
BMI, mean ± DS	24.2 ± 3.7	23.8 ± 2.8	0.71
Clinical characteristics			0.28
Laterality, n° (%)			
Right	7 (41.2)	11 (57.9)	
Left	10 (58.8)	8 (42.1)	
NRS, mean ± DS	7.6 ± 0,93	7.5 ± 1,21	0.79
Anatomical site of the fracture n° (%)			0.56
Pertrochanterics	10 (58.8)	12 (63.2)	
Intertrochanteric	2 (11.7)	1 (5.2)	
Subtrochanteric	5 (29.5)	6 (31.6)	
Type of surgery, n°(%)			0.62
Intramedullary nail	13 (76.5)	12 (63.2)	
Plate and screws	4 (23.5)	7 (36.8)	
Comorbidity, n° (%)			0.44
None	0 (0)	0 (0)	
1–2	3 (17.6)	5 (26.3)	
≥ 3	14 (82.4)	14 (73.7)	
Vitamin D, mean ± DS	17.7 ± 6.21	19.3 ± 5.41	0.41
Magnesemia levels, mean ± DS	1.86 ± 0,13	1.88 ± 0.21	0.73

Tables 3 and 4 show the trends of the variables analyzed in the control and treatment groups T0 to T1, respectively. In the control group, only the NRS scale showed significant improvements at T1 (7.5 ± 1.21 vs. 6.2 ± 1.09; *p* < 0.05), while none of the variables considered showed significant changes at the end of the rehabilitation protocol consisting of functional rehabilitation alone. On the contrary, in the treatment group we observed statistically significant changes in the magnesemia values (1.86 ± 0.13 vs. 2.11 ± 0.15; *p* < 0.05), NRS scale (7.6 ± 0.93 vs. 5.7 ± 0.81; *p* < 0.05), Tinetti test (14.2 ± 3.24 vs. 17.3 ± 1.15; *p* < 0.05) and Sarqol questionnaire (42.4 ± 8,.23 vs. 47.3 ± 5.21; *p* < 0,05). No statistically significant change was reported for vitamin D values. In addition, no adverse events were reported and adherence to the treatment was maximum.

**Table 3:** Effects of sucrosomal magnesium supplementation combined with therapeutic exercise in treatment group at T1

Characteristics	T0	T1	*p *value
Magnesemia, mean ± DS	1.86 ± 0.13	2.11 ± 0.15	< 0.05*
Vitamin D, mean ± DS	17.7 ± 7.51	21.3 ± 6.83	0.14
NRS, mean ± DS	7.6 ± 0.93	5.7 ± 0.81	< 0.05*
Tinetti test, mean ± DS	14.2 ± 3.24	17.3 ± 1.15	< 0.05*
SarQoL, mean ± DS	42.4 ± 8.23	47.3 ± 5.21	0.05*

*Significant improvement

**Table 4:** Effects of only therapeutic exercise in the control group at T1

Characteristics	T0	T1	*p *value
Magnesemia, mean ± DS	1.88 ± 0.21	1.94 ± 0,11	0.27
Vitamin D, mean ± DS	19.3 ± 5.41	21.7 ± 3.21	0.10
NRS, mean ± DS	7.5 ± 1.21	6.2 ± 1.09	< 0.05*
Tinetti test, mean ± DS	13.1 ± 4.52	15.2 ± 2.98	0.10
SarQoL, mean ± DS	44.6 ± 6.68	44.9 ± 5.54	0.88

*Significant improvement

By applying a classical linear regression model we compared the results obtained in patients in the treatment group with those of the control group (Table 5).

**Table 5:** Comparison of resultsat T1 between treatment group and control group

Characteristics	Treatment group	Control group	*p *value (control vs. treatment)
Magnesemia, mean ± DS	2.11 ± 0.15	1.94 ± 0.11	< 0.05
Vitamin D, mean ± DS	21.3 ± 6.83	21.7 ± 3.21	0.82
NRS, mean ± DS	5.7 ± 0.81	6.2 ± 1.09	0.02
Tinetti test, mean ± DS	17.3 ± 1.15	15.2 ± 2.98	0.01
SarQoL, mean ± DS	47.3 ± 5.21	44.9 ± 5.54	< 0.05

Comparing the treatment and control groups, we observed in the first a significant improvement in magnesaemia levels at T1 (2.11 ± 0.15 vs. 1.94 ± 0.41 mg/dl; *p* < 0.05) (Table 5), with percentage changes from baseline, 13.4% in the treatment group and 3.2% in the control group.

Regarding the vitamin D values, no statistically significant improvement was observed between the two groups (21.3 ± 6.83 vs. 21.7 ± 3.21; *p* = 0.82).

Analyzing the mean values of the NRS scale of both T1 groups, we noticed a significant improvement in pain in the patients belonging to the treatment group compared to those of the control group (5.7 ± 0.81 vs. 6.2 ± 1.09; *p* < 0.05).

For Tinetti test score, at T1 the treatment group obtained an average score higher than the control group (17.3 ± 1.15 vs. 15.2 ± 2.98; *p* = 0.001).

Finally, we also evaluated how the quality of life in relation to sarcopenia could vary after a month of integration with Sucrosomial®Magnesium. At T1, patients in the treatment group had a higher Sarqol score than the control group which remained substantially unchanged (47.3 ± 5.21 vs. 44.9 ± 5.54; *p* < 0.05).

By applying Pearson’s correlation we observed a moderate correlation between magnesemia values and Tinetti test result (*r* = 0.506) and between magnesium values and NRS scale (*r* = 0.618). While a strong correlation was observed between magnesemia values and the result of the SarQoL questionnaire (*r* = 0.849).

## Discussion

The fragility fracture of the proximal portion of the femur has an important clinical and economic impact on patients. It almost always requires hospitalization, in 20% of cases causes death, 50% of cases cause permanent disability and only 30% of cases heal completely [25, 26].

The first objective of this study was to assess the incidence of subclinical hypomagnesemia in a cohort of geriatric patients with a lateral fracture from the fragility of the proximal portion of the femur and their possible correlation with serum magnesium levels.

We showed that hypomagnesemiais found in half of the patients suffering from femur fracture from fragility, with a greater incidence of the subclinical form. For this reason, it would be desirable to include it among the modifiable risk factors for osteoporosis.

This requires greater attention in the diagnosis and correction of subclinical hypomagnesemia and not only that frank, clinically evident.

The aim of the second part was to assess the effectiveness of magnesium supplementation the outcome of osteoporotic patients.

Our study highlights the safety of Sucrosomial^®^Magnesium therapy demonstrated by adherence to treatment due to the absence of known side effects related to conventional magnesium (mainly gastrointestinal), single-dose formulation, and ease of intake.

In accord to our data, Brilli et al. [20] compared to the various magnesium formulations available on the market, the sucrosomial Magnesium formulation used, thanks to the presence of a phospholipid matrix around the magnesium ions, improves intestinal absorption of magnesium. Furthermore, this formulation, reaching the bloodstream directly without interacting with the intestinal mucosa, reduces the gastrointestinal side effects usually associated with Magnesium supplementation.

Another important point to highlight is that the correction of subclinical hypomagnesemia has positively influenced posture and balance as evidenced by the scale of Tinetti. Normally an unstable posture and altered pace pattern are both associated with an increased risk of falls and consequently fractures and are often associated with sarcopenia [27]. At the end of the study protocol, patients who received Sucrosomial®Magnesium supplementation also showed an improvement in the score of the Sarqol questionnaire, especially in the mental and physical health domain. All this determines an improvement in the quality of life in relation to sarcopenia, as a consequence of the improvement in the functional capacity of the patients which led to their greater participation in daily life and recreational activities. The improvement of balance and strength is related to a reduction in the risk of falls and refractions.

However, in the past WHI studies have tried to analyze the possible relationship between magnesemia and the risk of falls. Although possible, the authors did not conclude for an observed possible direct correlation between magnesium and increased risk of falls probably. It has been hypothesized that the falls may be the result of the fact that women were more active and therefore have greater potential to fall and fracture [28].

The results obtained in the second part of the study should not be surprising, taking into account the extensive literature on the effects of magnesium and the increasing amount of evidence on the topic of subclinical hypomagnesemia.

Indeed, studies [9–16] have shown that the average dietary intake of magnesium of the European and North American population is lower than the recommended daily intake, exposing, in the long run, the risk of chronic magnesium deficiency.

The roles of magnesium in the determination of both sarcopenia and osteopenia, as well as in various mitochondrial processes involved in the production of ATP are also evident [19–29].

The importance of magnesium in the muscles is underlined by the fact that one of the largest deposits of magnesium in our body is represented by the muscle [30]. Magnesium, in addition to being involved in muscle metabolism processes, such as protein and ATP synthesis and the degradation of glycogen, also influences muscle performance through energy metabolism, transmembrane transport, and muscle contraction and relaxation [19].

The role of magnesium as a modifiable risk factor for osteoporosis is now known, in consideration of the fact that low magnesium levels lead to reduced osteoblastic and osteoclastic activity, bone fragility, and strength or reduction of vitamin D and PTH [31, 32].

In the literature, substantial improvements in total body BMD have been shown following the intake of magnesium, describing a positive association [33]. In the Framingham Heart Study, a 2% higher trochanteric BMD has observed for every 100 mg of Mg consumed [34]. Further studies have shown an improvement in BMD or a reduction in markers of bone turnover following the intake of magnesium, however, the relationship with the outcome of the fracture was not clear [35].

Veronese et al. have demonstrated that the intakeof 300 mg/day of magnesium hydroxide has brought an improvement in the physical performance of treated subjects, especially in terms of gait speed and chairs to stand test [36].

Our study is in line with this, in fact the results of the Sarqol questionnaire show a clear improvement in the functional capacity and participation of the patients examined.

An interesting fact highlighted by our study was the greater reduction of pain in patients undergoing the integration with Magnesium Sucrosomiale. Although this may be a result of several factors, including the therapeutic exercise that contributes to the control of pain, in the literature it is now known the role of Magnesium in the control of pain. Although it has no direct antinociceptive effects, it is considered a natural calcium antagonist. By inhibiting NMDA receptors, it blocks the entry of calcium ions into the cell, performing an analgesic effect and thus avoiding the development of central sensitization and receptor hypersensitivity [37].

Our study further emphasizes the concept that osteoporosis is increasingly considered a condition of Sarco-osteopenia, thus being able to define a pathology with two protagonists (sarcopenia and osteopenia) that, with the advancement of time and in the absence of a holistic and multidisciplinary approach, determines the so-called “spiral of fragility”. Magnesium, thanks to its dual and synergic role both on bone and muscle metabolism, is particularly suitable for this situation. It is therefore suggested to underline the importance of assessing the magnesium status and the correction of hypomagnesemia of elderly subjects, as it can improve their rehabilitation outcome. The correct dietary intake or direct supplementation of magnesium improves muscle strength as well as reducing the incidence of symptoms such as myalgia and muscle cramps, resulting in greater compliance and a better rehabilitation result, especially in a critical moment such as the immediate post-operative.

### Study limitations

The main limitations of our study were the small sample size, which does not allow us to generalize the results obtained and the inability to determine, even roughly, the average dietary intake of magnesium of individual patients. About this, the variations in magnesemia values could be the result not only of the integration practiced but also of a higher dietary intake arbitrarily taken by the patient.Another limitation, due to the low sample size, was that it did not stratify magnesemia levels and brittle fractures with other risk factors.

Possible suggestions for future studies could be a more precise analysis, for example through the dosage of carboxylatedosteocalcin, of the effects of magnesium integration on osteogenesis in the post-fracture period. Again, it might be interesting to evaluate the organic status of magnesium with more specific analytical methods, such as the determination of the ionic form or intra-erythrocyte quantity, thus assessing the actual correlation with the total magnesemia parameter. Finally, it may be useful to evaluate, through the analysis of bone fragments taken during osteosynthesis/prosthetization, the amount of cortical magnesium true reservoir of this ion, compared to serum magnesemia, to shed light on more loyal subclinical hypomagnesemia suspect criteria.

## Conclusion

The assessment of magnesemia, in elderly patients at risk of osteo-sarcopenia, should be carried out with routine humoral examinations and involve all specialists who treat the health of the elderly patient. Therefore, it would be desirable to measure magnesemia and correct any hypomagnesemia conditions immediately, given the health benefit and low cost.

The results of our study, albeit obtained on a small sample, seem to demonstrate that magnesium supplementation positively influences posture and balance and improves the quality of life of subjects with frank or subclinical hypomagnesemia. In general, adding magnesium even to the habits of the healthy elderly population could improve bone and muscle metabolism, and consequently allow better rehabilitation results and reduce the risk of falls and fractions.

## Data Availability

The datasets generated during and/or analyzed during the current study are available from the corresponding author on reasonable request.
